# A plant-microbe treaty: coordinated action of plant and microbial proteins strengthens immunity in apple

**DOI:** 10.1093/plphys/kiag156

**Published:** 2026-03-19

**Authors:** Arijit Mukherjee

**Affiliations:** Howard Hughes Medical Institute and Department of Biology, University of North Carolina at Chapel Hill, Chapel Hill, NC 27599-3280, United States

Plants are colonized by a diverse community of microbes comprising both pathogenic and beneficial ones (Trivedi et al. 2020). The latter can protect the host plant from pathogen attacks and function as biocontrol agents. These microbes suppress pathogens either via direct inhibition or by indirect activation of the plant immune system (induced resistance). For instance, the plant-associated microbial communities produce diverse antibiotics to deter pathogens, and the well-known *Pseudomonas simiae* WCS417 can induce systemic resistance against a broad range of pathogens in Arabidopsis, tomato, and other plants (Pieterse et al. 2014; Getzke et al. 2023; Mukherjee et al. 2023).

Recognition of pathogen-secreted effectors activates a strong immune response in plants (Jones et al. 2024). This effector-triggered immunity is relatively well characterized; however, the molecular mechanism(s) of induced resistance via beneficial bacteria secreted effector(s) remain poorly understood. In this issue of *Plant Physiology*, Yu and colleagues revealed one such mechanism wherein a secreted effector from the previously known biocontrol bacterium *Saccharothrix yanglingensis* (Sy) activates the immune system in apple (Malus domestica, Md) to confer resistance against the devastating Valsa canker (AVC) pathogen *Valsa mali* (Yan et al. 2012; Yu et al. 2026).

Using a combination of bioinformatic screening, heterologous expression, and functional and localization assays, the authors identified a highly conserved cupredoxin like protein (CD1) as the most potent secreted effector from Sy delivered inside plant cells. The immune activating role of SyCD1 was then tested using multiple approaches via its transient and stable overexpression in tobacco and multiple apple varieties. Consistent with typical immune induction, overexpression of SyCD1 led to reactive oxygen species (ROS) accumulation, callose deposition, and a reduced lesion area against the AVC pathogen. Moreover, reverse transcription quantitative PCR analyses and hormone biosynthesis inhibition assays confirmed jasmonic acid (JA) signaling as the primary pathway involved in SyCD1 mediated resistance.

To identify host proteins acting downstream of SyCD1, the authors employed pull-down MS and yeast-2-hybrid assays. These complementary approaches identified MdSP1, a truncated version of MdCML13 (probable calcium binding protein), as a candidate interactor of SyCD1. Physical interaction between SyCD1 and MdSP1 was subsequently validated by co-immunoprecipitation and subcellular localization assays in tobacco leaves, which revealed their co-localization at the cell membrane. Interestingly, both mutual and individual overexpression of MdSP1 and SyCD1 revealed their synergistic role in typical immune induction such as callose deposition and ROS accumulation with consequent reduction of lesion area following infection with the AVC pathogen. Further, stable overexpression and RNA-interference lines, together with transcriptome profiling, established MdSP1 as a positive regulator of immunity in apple. Since MdSP1 promoter harbored JA responsive cis-elements, the authors developed luciferase and GUS reporter lines to investigate the involvement of JA signaling. Treatments with the JA analog methyl-jasmonate, the JA biosynthesis inhibitor ibuprofen, and SyCD1 protein in these lines collectively demonstrated that SyCD1 induces MdSP1 expression through JA signaling.

The authors further delved to identify proteins acting downstream of MdSP1 by adopting a similar approach above. These analyses identified MdANK, an ankyrin repeat containing protein, as the interacting partner of MdSP1, without the evidence of formation of a ternary MdSP1-SyCD1-MdANK complex. MdANK induction and its homodimerization were regulated via SyCD1 induced JA signaling, supporting its role as a positive regulator of immunity in apple. RNA-seq and reverse transcription quantitative PCR assays further revealed an intersection of MdSP1- and MdANK-mediated immunity via MAP kinase (Mitogen associated protein) signaling pathway. Consistent with this finding, transient coexpression of MdSP1 and MdANK in tobacco resulted in enhanced accumulation of phosphorylated MPK6. Importantly, their coexpression elicited significantly stronger immune responses, including elevated callose deposition and ROS bursts, compared with individual expression of either protein. Together, these results indicate that MdANK amplifies MdSP1-dependent signaling to potentiate MAPK activation in response to SyCD1.

The effectiveness of the SyCD1-MdSP1-MdANK module presented in this article indicates an example of cross-kingdom partnership in bolstering immunity in apple (Fig. 1). Since SyCD1 does not physically interact with MdANK, the direct mechanism of MdANK homodimerization remains to be further investigated (Fig. 1). Given the environmental repercussions and limited penetration of fungicides inside xylem and phloem vessels to deter the AVC pathogen, the indirect mechanism of effector-mediated immune activation offers a more effective and sustainable solution to managing this disease. Additionally, the coordinated action of both host and microbe-derived proteins highlights a promising intervention point to improve the efficacy of biocontrol applications.

**Figure 1:**
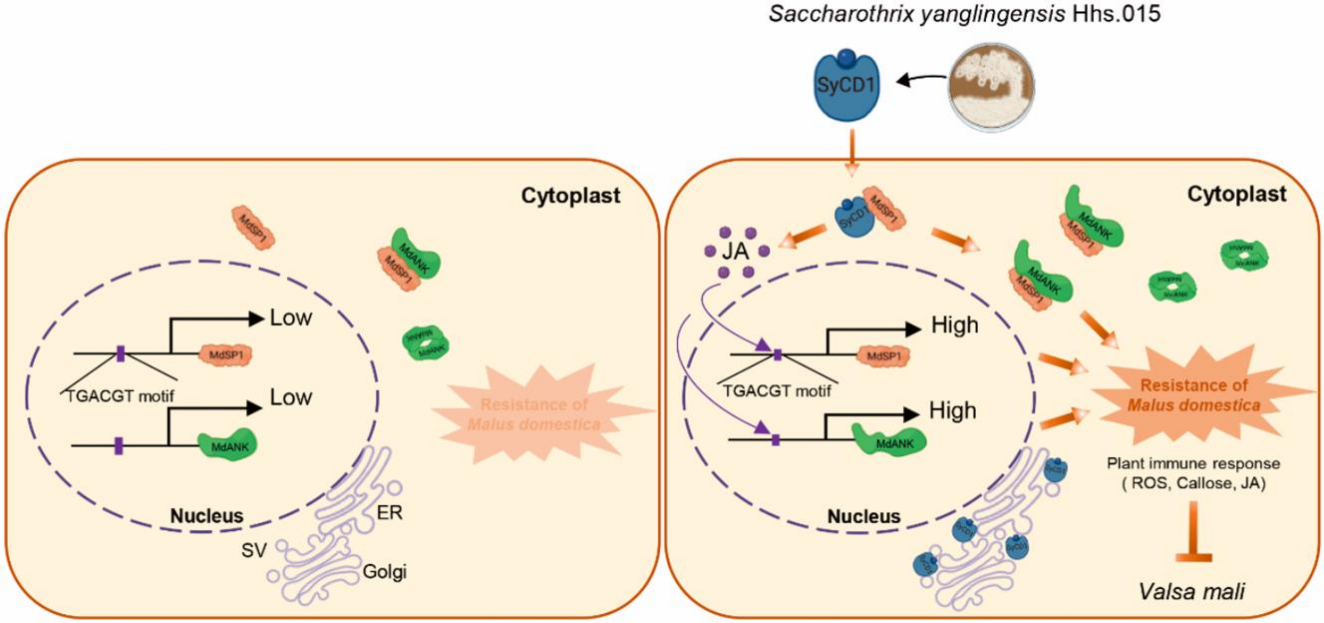
The coordinated action of SyCD1, MdSP1, and MdANK enhances resistance in apple (Malus domestica) against Valsa canker pathogen *V. mali*. *Saccharothrix yanglingensis* (Sy) secretes the effector SyCD1 inside host cells and induces MdSP1 and MdANK expression via JA signaling. SyCD1 interacts with MdSP1 and induces homodimerization of MdANK. The coordinated actions of these proteins where SyCD1 acts as the initiator and MdSP1-MdANK module acts as the amplifier, presents a multi-layered cross-kingdom protection mechanism against the AVC pathogen, *V. mali*. Figure is taken from Yu et al. (2026).

## Related articles in *Plant Physiology*:

Thomazella et al. (2025) reviewed the role of plant-associated microbial communities in protection against pathogens, also highlighting the role of their secreted effectors in modulation of plant immunity.

Wang et al. (2024) reported a plant-derived peptide playing a crucial role in systemic immunity in Arabidopsis

## Data Availability

Not applicable.
